# Sex Differences in Perceptions and Behavioral Context of Electronic Cigarette Use: A Cross-Sectional Study in Jeddah, Saudi Arabia

**DOI:** 10.3390/ijerph23070848

**Published:** 2026-06-29

**Authors:** Shada Alodaini, Mohammed Ahmed Alzahrani, Mohammed Awad Algarni, Wajdi Hussain Alamri, Ahmed H. Alasimi, Faisal A. Turkestani, Alaa Bugis, Jameel Hakeem

**Affiliations:** 1 Department of Respiratory Therapy, College of Applied Medical Sciences, King Saud Bin Abdulaziz University for Health Sciences, Jeddah 21423, Saudi Arabia; 421120227@ksau-hs.edu.sa (M.A.A.); 421120236@ksau-hs.edu.sa (M.A.A.); 421120299@ksau-hs.edu.sa (W.H.A.); turkestanif@ksau-hs.edu.sa (F.A.T.); bugisa@ksau-hs.edu.sa (A.B.); hakeemj@ksau-hs.edu.sa (J.H.); 2 King Abdullah International Medical Research Center, Jeddah 22384, Saudi Arabia; 3 Ministry of the National Guard-Health Affairs, Jeddah 23462, Saudi Arabia; 4 National Heart and Lung Institute, Imperial College London, London SW7 2AZ, UK; a.alasimi25@imperial.ac.uk

**Keywords:** e-cigarettes, vaping, gender differences, risk perception, social norms, media influence

## Abstract

**Highlights:**

**Public health relevance—How does this work relate to a public health issue?**

- Electronic cigarette use is rapidly increasing among young adults in Saudi Arabia, yet sex-specific behavioral and perceptual differences remain poorly understood.
- This study addresses critical public health concerns related to vaping normalization, risk perception, and social media influence within a rapidly evolving sociocultural environment.

**Public health significance—Why is this work of significance to public health?**

- Males demonstrated consistently higher endorsement of vaping-related utility, social acceptability, and marketing influence across multiple behavioral domains.
- The findings identify important sex-based disparities in vaping perceptions that may contribute to differential uptake and future nicotine dependence among young adults.

**Public health implications—What are the key implications or messages for practitioners, policy makers and/or researchers in public health?**

- Public health interventions targeting vaping among Saudi youth should incorporate sex-specific prevention strategies addressing perceived benefits and social media exposure.
- Regulatory policies and educational campaigns should focus on correcting harm misperceptions and limiting youth-targeted vaping marketing in emerging vaping contexts.

**Abstract:**

**Importance** Understanding sex-based differences in vaping-related perceptions and behaviors is critical for informing targeted public health interventions, particularly in regions where e-cigarette use is emerging. **Objective** To examine sex differences across multiple vaping-related domains, including behavioral context, perceived utility, risk perception, social norms, and media influence among young adults in Saudi Arabia. **Design, Setting, and Participants** This cross-sectional study was conducted among 388 young adults aged 18–24 years in Jeddah, Saudi Arabia. Data were collected using a structured, theory-informed questionnaire adapted from established tobacco-use instruments. **Exposure** Sex (male vs. female), monthly income, and marital status. Main Outcomes and Measures Domain-specific scores were derived from 12 vaping-related items grouped into 5 conceptual domains. Scores were categorized into low, medium, and high levels using tertiles. Associations were assessed using ordinal logistic regression. **Results** Among 388 participants, 228 (58.8%) were male and 160 (41.2%) were female. Males consistently demonstrated higher levels of endorsement across domains. In adjusted models, females had significantly lower odds of higher domain scores across situational use (OR, 0.62; 95% CI, 0.45–0.86), perceived utility (OR, 0.58; 95% CI, 0.41–0.81), risk perception (OR, 0.54; 95% CI, 0.39–0.75), social norms (OR, 0.63; 95% CI, 0.45–0.88), and media influence (OR, 0.49; 95% CI, 0.35–0.69). Lower income was also associated with reduced endorsement across multiple domains. **Conclusions and Relevance** Significant sex-based differences exist in vaping-related perceptions and behaviors among young adults. These findings highlight the need for sex-specific prevention strategies addressing perceived benefits and marketing exposure in emerging vaping contexts.

## 1. Introduction

Electronic cigarette (e-cigarette) use has emerged as a rapidly expanding public health concern globally, particularly among young adults and urban populations [1,2]. In Saudi Arabia, the adoption of vaping has accelerated over the past decade, driven by aggressive marketing, perceived harm reduction compared with conventional cigarettes, and increasing product accessibility [3,4]. Recent studies suggest that e-cigarette use prevalence among young adults in Saudi Arabia ranges widely from approximately 10% to over 30% in university populations reflecting both rapid uptake and heterogeneity across settings [5,6]. These patterns are further compounded by substantial misperceptions regarding safety, with many individuals viewing vaping as less harmful despite growing evidence linking e-cigarette use to respiratory and cardiovascular risks.

Despite this growing body of evidence, important epidemiologic gaps remain. First, the Saudi literature has largely focused on prevalence estimates [6] or general knowledge and attitudes [7], often within convenience samples such as university students, with limited exploration of behavioral, social, and perceptual determinants across multiple domains. Second, and more critically, there is a paucity of research examining sex-based differences in vaping-related perceptions and behaviors within the Saudi context. Existing studies consistently report higher vaping prevalence among males, yet emerging evidence suggests increasing uptake among females, particularly in urban centers [8]. However, these studies rarely examine how perceptions, social norms, and behavioral contexts differ by sex, nor do they incorporate multidimensional frameworks that capture these constructs comprehensively.

Understanding sex differences in vaping behavior is particularly important in Saudi Arabia given the profound sociocultural and policy shifts occurring under Vision 2030. National reforms aimed at enhancing women’s participation in education, employment, and public life have substantially transformed gender roles and social environments [9]. These changes have been accompanied by increased autonomy, mobility, and consumer engagement among Saudi women, including participation in previously male-dominated spaces [10]. Within this evolving context, health behaviors including tobacco and nicotine use are also undergoing transition. The intersection of female empowerment and exposure to new consumer markets, including vaping products, raises critical questions regarding changing risk behaviors and perceptions.

Simultaneously, the vaping market in Saudi Arabia has expanded rapidly, particularly in major urban centers such as Jeddah and Riyadh [11]. The legalization and regulation of e-cigarette sales have facilitated widespread availability through retail outlets and online platforms, making vaping products easily accessible [12]. In addition, the proliferation of specialized vape shops, flavored products, and social media marketing has normalized vaping in certain social settings, especially among younger populations. Prior research has demonstrated that exposure to marketing and peer behaviors plays a significant role in shaping vaping initiation and continuation, yet these influences may differ substantially by sex due to cultural norms and social expectations [13].

Another key concern is the ambiguity surrounding the health risks of vaping within the Saudi population. Recent evidence indicates that dual use of conventional cigarettes and e-cigarettes is increasingly common among adolescents and young adults in Saudi Arabia, raising concerns about sustained nicotine dependence rather than complete substitution of combustible tobacco products [14]. Furthermore, many users report initiating e-cigarette use as a smoking cessation aid or as a perceived less harmful alternative to conventional smoking, although long-term dual use remains prevalent among a substantial proportion of users [15].

Taken together, these factors underscore the need for a more nuanced understanding of vaping behavior in Saudi Arabia that extends beyond prevalence estimates to incorporate behavioral context, perceived utility, and media influence. Importantly, examining these domains through a sex-based lens is essential to identify differential patterns that may inform targeted public health interventions. Accordingly, this study aimed to evaluate sex differences in vaping-related perceptions, social norms, and behavioral context across multiple domains using a structured framework informed by the Population Assessment of Tobacco and Health (PATH) [16] study and the Theory of Planned Behavior [17]. By integrating multidimensional assessment with robust statistical modeling, this study seeks to address a critical gap in the Saudi literature and provide evidence to inform gender-responsive tobacco control strategies in the context of a rapidly evolving social and regulatory environment.

## 2. Methods

### 2.1. Study Design and Setting

This cross-sectional observational study was conducted to evaluate sex differences in vaping-related perceptions, behavioral context, and influencing factors among young adults in Jeddah, Saudi Arabia. Data were collected from 20 May 2025 to 10 December 2025 using a structured, self-administered electronic questionnaire distributed through online platforms, including social media networks and institutional channels.

The survey was distributed electronically using a web-based platform and disseminated through multiple digital channels to maximize reach within the target population. In Jeddah, internet access and smartphone ownership are highly prevalent among young adults, which may have facilitated equitable access to the online survey across the target population. The questionnaire link was shared via commonly used social media platforms, including WhatsApp, X (formerly Twitter), and Instagram, as well as through institutional and peer networks within universities and community groups in Jeddah. Participants were encouraged to share the survey within their networks (snowball distribution) to increase participation among eligible individuals. No financial incentives were provided. To reduce duplicate responses, the survey platform restricted multiple submissions from the same device where feasible. Participation was voluntary, and respondents were able to complete the questionnaire anonymously at their convenience. A pilot study was conducted among 5 participants from the target population to assess the clarity, comprehensibility, and cultural appropriateness of the questionnaire items. Minor wording refinements were made based on participant feedback to improve readability and ensure consistent interpretation prior to full-scale data collection. The study design and reporting adhered to the Strengthening the Reporting of Observational Studies in Epidemiology (STROBE) guidelines [18].

### 2.2. Study Population and Sampling Strategy

The study population comprised young adults aged 18 to 24 years residing in Jeddah who reported prior experience with electronic cigarette use. Eligibility criteria included age within the defined range, residence in Jeddah, and ability to complete the electronic survey. Participants who did not meet these criteria or submitted incomplete questionnaires were excluded. A non-probability snowball sampling approach was employed, whereby all eligible participants who accessed and completed the survey during the study period were included. This approach was selected due to the absence of a defined sampling frame for vape users and is consistent with epidemiologic practice in behavioral survey research involving hard-to-reach populations.

### 2.3. Sample Size Determination

The required sample size was estimated for a cross-sectional survey designed to measure vaping-related perceptions and behavioral domains among young adults. Because no single prior estimate was available for all study domains, the sample size calculation used the conservative assumption of 50% response distribution, which produces the maximum required sample size for proportion-based survey estimates. Using a 95% confidence level, 5% margin of error, and an assumed source population of approximately 20,000 young adults, the minimum required sample size was estimated at 377 participants. To account for incomplete responses and ensure adequate precision for sex-stratified and multivariable analyses, the target recruitment exceeded the minimum estimate. A total of 392 participants completed the questionnaire and were included in the primary analysis. For domain-based analyses requiring complete responses across all relevant survey items, complete-case analysis was performed.

### 2.4. Survey Instrument, Domain Development, and Scoring

The questionnaire was adapted from domains used in the Population Assessment of Tobacco and Health Study and prior tobacco perception literature, with modifications to reflect the Saudi sociocultural context and the study objective of evaluating sex differences in vaping-related perceptions and behavioral context [19,20,21,22]. The instrument included demographic items and 12 vaping-related items organized a priori into 5 conceptual domains based on behavioral theory and tobacco-use literature. The Situational Use and Behavioral Context domain included items assessing use of vapes in places where cigarette smoking is not allowed and vaping while spending time with others. The Perceived Utility and Functional Benefits domain included beliefs that vapes help people quit smoking cigarettes, feel similar to cigarettes, and are affordable. The Risk Perception and Sensory Evaluation domain included perceived lower harm compared with cigarettes, unpleasant smell, and availability of preferred flavors. The Social Acceptability and Norms domain included perceived acceptability of vaping among non-smokers and influence from important people. The Media and Marketing Influence domain included exposure to social media advertising and celebrity influence.

Each item was coded as a binary response, with endorsement of the vaping-related perception, behavior, or influence coded as 1 and non-endorsement coded as 0. Domain scores were calculated by summing item responses within each domain, so higher scores indicated greater endorsement of that domain. Because the number of items differed across domains, domain scores were interpreted within domain rather than directly compared as raw totals across domains. For categorical analysis, each domain score was classified into low, medium, or high levels using empirical tertile-based thresholds. When the score distribution did not support 3 distinct categories because of limited score range or tied values, categories were collapsed or interpreted according to the observed distribution. This approach was used because no validated cut-points exist for these adapted vaping perception domains. Internal consistency reliability was assessed for each multi-item domain using Cronbach α, with values of 0.70 or higher considered acceptable for exploratory behavioral research. Domains with lower α were interpreted as formative behavioral constructs rather than reflective psychometric scales.

### 2.5. Domain Classification

Domain scores were categorized into low, medium, and high levels using an empirical distribution–based approach. Specifically, tertile cutoffs (33rd and 66th percentiles) were used to classify participants into three approximately equal groups within each domain [23]. This method allows relative ranking of individuals based on their position within the observed distribution and is commonly applied in epidemiologic and behavioral research when validated or clinically established thresholds are not available. Tertile-based categorization has been widely used in studies examining health behaviors, perceptions, and composite indices to facilitate interpretation and enable ordinal regression modeling [24]. Because the domains in this study were derived from adapted survey constructs rather than validated psychometric scales with predefined cut-points, a data-driven approach was considered appropriate to avoid imposing arbitrary thresholds. This approach is consistent with prior epidemiologic practice in cross-sectional analyses of behavioral constructs and composite scores, where categorization based on quantiles (e.g., tertiles or quartiles) is used to capture gradients of exposure or perception while preserving statistical power. In domains with limited score variability or tied values, category boundaries were examined and adjusted pragmatically to maintain interpretability while avoiding empty or unstable categories. Sensitivity of classification was assessed by inspecting score distributions across sex strata to ensure consistent categorization.

### 2.6. Statistical Analysis

All analyses were performed after data screening for completeness, coding errors, duplicate entries, and internal consistency. Respondents with missing values in variables required for domain scoring or regression modeling were excluded from complete-case analyses. Descriptive statistics were used to summarize participant characteristics and survey responses. Categorical variables were reported as frequencies and percentages. Domain scores were summarized using medians and interquartile ranges because the scores were ordinal and bounded. Sex differences in domain classifications were assessed using Pearson χ^2^ tests; when expected cell counts were small, exact or likelihood-ratio approaches were considered. Effect estimates were reported with 95% confidence intervals where appropriate.

Ordinal logistic regression was used to examine the association between sex and ordered domain classification levels, defined as low, medium, and high. Models included sex as the primary exposure and adjusted for monthly income and marital status because these variables may influence vaping-related perceptions, affordability, and social context. The proportional odds assumption was assessed for each ordinal model; if the assumption was not met, findings were interpreted cautiously or alternative binary contrasts were considered. Multicollinearity among covariates was evaluated using variance inflation factors and cross-tabulation of sparse categories. Model fit and influential observations were assessed using standard diagnostic procedures. Odds ratios greater than 1 indicated higher odds of being in a higher domain classification category, whereas odds ratios less than 1 indicated lower odds. For selected individual binary survey items, binary logistic regression was used to estimate sex-specific and income-related associations with vaping-related perceptions and influences. Results were reported as odds ratios with 95% confidence intervals and 2-sided *p* values. Statistical significance was defined as *p* < 0.05. Given the exploratory nature of the domain-specific analyses, results were interpreted with emphasis on effect size, direction, and consistency across domains rather than statistical significance alone. Analyses were conducted using Python (version 3.13.5, Beaverton, OR, USA) with pandas 2.2.3, NumPy 2.3.5, statsmodels 0.14.6, scikit-learn 1.8.0, SciPy 1.17.0, and Matplotlib 3.10.8. Excel and Word outputs were generated using openpyxl 3.1.5 and python-docx 1.2.0, respectively.

### 2.7. Variable Definitions and Classification

Domain scores were calculated by summing item responses within each domain, with higher scores indicating greater endorsement of the construct. To facilitate analysis, domain scores were categorized into low, medium, and high levels using tertile-based cutoffs, consistent with epidemiologic practice when validated thresholds are not available. The primary exposure variable was sex, and secondary covariates included monthly income and marital status.

### 2.8. Ethical Considerations

The study was approved by the Institutional Review Board of King Abdullah International Medical Research Center (KAIMRC; IRB/2106/23). Participation was voluntary, and informed consent was obtained electronically from all participants prior to survey completion. No personally identifiable information was collected, and all data were anonymized and handled in accordance with ethical standards and the Declaration of Helsinki.

## 3. Results

### 3.1. Sex Differences in Vaping-Related Domains and Participant Characteristics

**Figure 1:**
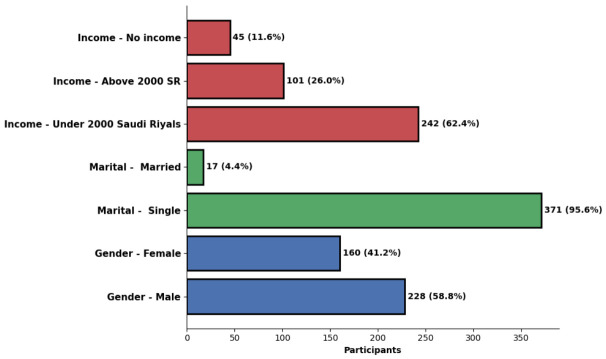
Participant Characteristics.

Among 388 participants, 228 (58.8%) were male and 160 (41.2%) were female. Significant sex differences were observed across multiple vaping-related domains. In the Situational Use and Behavioral Context domain, males were more likely than females to report higher levels of use, with a greater proportion classified as high (61 [15.7%] vs. 34 [8.8%]) and medium (43 [11.1%] vs. 17 [4.4%]), whereas females were more frequently classified in the low category (109 [28.1%] vs. 124 [32.0%]; *p* = 0.016). In the Perceived Utility and Functional Benefits domain, males demonstrated higher endorsement of vaping-related benefits, with greater proportions in the medium (103 [26.5%] vs. 49 [12.6%]) and high categories (39 [10.1%] vs. 19 [4.9%]), while females were more commonly represented in the low category (92 [23.7%] vs. 86 [22.2%]; *p* < 0.001) (Figure 1, Table 1).

Similarly, in the Risk Perception and Sensory Evaluation domain, males were more likely to report higher levels of favorable perceptions toward vaping, with higher proportions in both medium (80 [20.6%] vs. 28 [7.2%]) and high categories (74 [19.1%] vs. 41 [10.6%]), whereas females were more frequently classified as low (91 [23.5%] vs. 74 [19.1%]; *p* < 0.001).

In the Social Acceptability and Norms domain, males again showed higher levels of endorsement, with greater proportions in the medium (71 [18.3%] vs. 36 [9.3%]) and high categories (20 [5.2%] vs. 7 [1.8%]), while females were more likely to report low levels of social acceptability (117 [30.2%] vs. 137 [35.3%]; *p* = 0.022).

In contrast, in the Media and Marketing Influence domain, females were more likely to report lower levels of influence (94 [24.2%] vs. 73 [18.8%]), whereas males were more frequently classified in the medium category (155 [39.9%] vs. 66 [17.0%]; *p* < 0.001), indicating greater susceptibility to marketing exposure among males. No significant sex differences were observed in monthly income distribution (*p* = 0.304). However, marital status differed between groups, with the majority of participants being single (371 [95.6%]), and a modest but statistically significant difference observed by sex (*p* = 0.024).

Overall, these findings demonstrate consistent sex-based differences across multiple behavioral and perceptual domains, with males exhibiting higher levels of situational use, perceived utility, favorable risk perceptions, and social acceptance of vaping, whereas females were more likely to report lower levels across these domains (Table 1).

### 3.2. Distribution of Vaping-Related Domains

**Figure 2:**
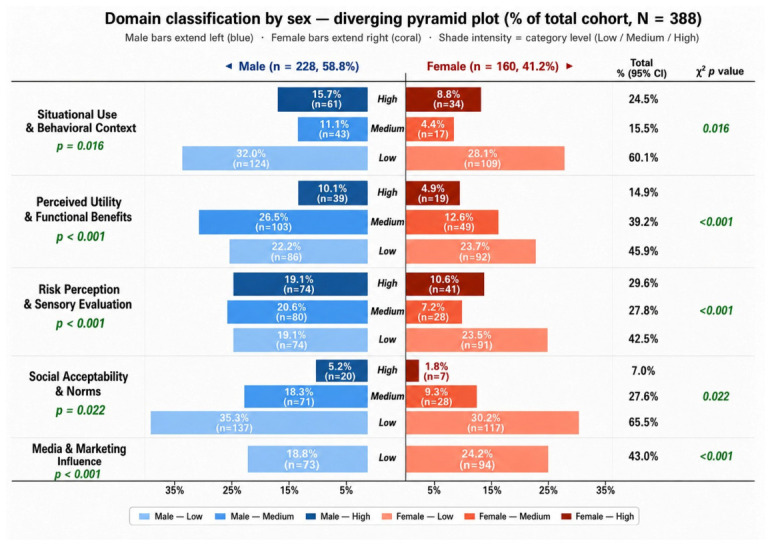
Sex Differences in Domain Classification of Vaping-Related Factors. Footnote: Male bars extend to the left and female bars extend to the right. Bar length represents the percentage of the total cohort within each sex and domain-classification category. Blue shades indicate male participants, and coral shades indicate female participants; darker shades represent higher endorsement categories. Domain classifications are ordered as low, medium, and high, except for Media and Marketing Influence, where only low and medium categories were available. The right-side columns show the total percentage for each category and the χ^2^ *p* value comparing sex distributions across categories within each domain.

Across 388 participants, most domains were skewed toward low or moderate levels. Situational use was predominantly low (233 [60.1%]), with 95 (24.5%) classified as high. Perceived utility was largely low to moderate (178 [45.9%] low; 152 [39.2%] medium), with fewer participants reporting high utility (58 [14.9%]). Risk perception showed greater variability (165 [42.5%] low; 115 [29.6%] high). Social acceptability was primarily low (254 [65.5%]), with only 27 (7.0%) classified as high. Media influence was mainly moderate (221 [57.0%]). Overall, vaping-related behaviors and perceptions were characterized by low situational use and social acceptance, with moderate variability in perceived utility, risk, and media influence (Table 2, Figure 2).

### 3.3. Multivariable Associations Between Participant Characteristics and Domain Classification

**Figure 3:**
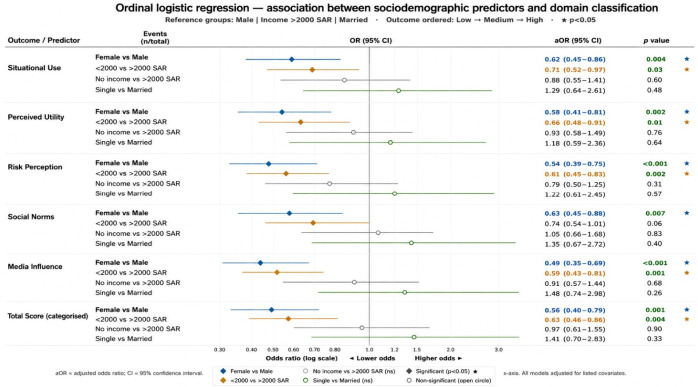
Multivariable Associations Between Participant Characteristics and Domain Classification. Footnote: Points represent adjusted odds ratios from ordinal logistic regression models, and horizontal lines represent 95% confidence intervals. The outcome was ordered from low to medium to high domain classification; odds ratios below 1 indicate lower odds of being in a higher domain category, whereas odds ratios above 1 indicate higher odds. Models were adjusted for sex, monthly income, and marital status. Reference groups were male sex, monthly income > 2000 SAR, and married status. Filled markers indicate statistically significant associations at *p* < 0.05, and open markers indicate non-significant associations. Exact estimates, confidence intervals, and *p* values are presented in Table 2.

In ordinal logistic regression analyses, female participants had consistently lower odds of higher domain classification across all domains compared with males, including situational use (OR, 0.62; 95% CI, 0.45–0.86; *p* = 0.004), perceived utility (OR, 0.58; 95% CI, 0.41–0.81; *p* = 0.002), risk perception (OR, 0.54; 95% CI, 0.39–0.75; *p* < 0.001), social norms (OR, 0.63; 95% CI, 0.45–0.88; *p* = 0.007), and media influence (OR, 0.49; 95% CI, 0.35–0.69; *p* < 0.001). Similarly, lower income (<2000 SAR) was associated with reduced odds of higher domain classification across most domains, including situational use (OR, 0.71; *p* = 0.032), perceived utility (OR, 0.66; *p* = 0.011), risk perception (OR, 0.61; *p* = 0.002), and media influence (OR, 0.59; *p* = 0.001), while the association with social norms did not reach statistical significance (*p* = 0.058). No consistent associations were observed for participants with no income or for marital status across domains. For the overall categorized score, females (OR, 0.56; 95% CI, 0.40–0.79; *p* = 0.001) and those with lower income (OR, 0.63; 95% CI, 0.46–0.86; *p* = 0.004) had significantly lower odds of higher overall domain classification (Figure 3).

## 4. Discussion

In this cross-sectional study of young adults in Jeddah, Saudi Arabia, we observed consistent and behaviorally meaningful sex-based differences across multiple vaping-related domains, including situational use, perceived utility, risk perception, social norms, and media influence. Males demonstrated higher levels of engagement and more favorable perceptions of vaping across nearly all domains, while females were more likely to report lower endorsement of vaping-related behaviors and influences. These findings extend the existing Saudi literature beyond prevalence estimates by demonstrating that sex differences are not limited to use patterns but are deeply embedded in behavioral context, perception, and social influence structures.

Our findings are consistent with previous studies from Saudi Arabia [4,25], the United Kingdom [26], and the United States [27], which reported a higher prevalence of vaping among males. This pattern has often been attributed to sociocultural norms that historically normalize tobacco use among men while discouraging it among women. However, the present study adds nuance by demonstrating that these differences persist across multiple behavioral and perceptual domains. For example, males were more likely to report higher perceived utility of vaping, including beliefs related to smoking cessation and affordability. Similar patterns have been reported in international studies, where males tend to endorse harm-reduction narratives and functional benefits of e-cigarettes more strongly than females [28]. This may reflect differential exposure to pro-vaping messaging and greater acceptance of risk-taking behaviors among men.

In contrast, females in this study were more likely to report lower levels of perceived utility, risk tolerance, and social acceptability, suggesting a more cautious perception profile. This aligns with global evidence indicating that women generally perceive greater health risks from tobacco products and are less likely to adopt novel nicotine delivery systems [29]. However, emerging literature suggests that this gap may be narrowing, particularly in urban settings and among younger cohorts, where changing gender norms and increased autonomy influence health behaviors [30]. Within the Saudi context, ongoing societal transformations under Vision 2030, including increased female participation in education and the workforce, may contribute to evolving patterns of nicotine use and perception.

The observed differences in the media and marketing influence domain are particularly notable. Males demonstrated significantly higher susceptibility to vaping-related marketing and social media exposure, whereas females were more frequently classified in lower influence categories. This finding contrasts with some Western countries, including Finland, Denmark, and Sweden, in which females are reported to be more responsive to social media marketing, particularly when linked to aesthetics and lifestyle branding [31]. The divergence observed in this study may reflect context-specific cultural norms, differential engagement with public spaces, or variations in targeted marketing strategies within Saudi Arabia. It also highlights the importance of considering local sociocultural dynamics when interpreting the impact of media exposure on health behaviors.

Another important finding is the overall distribution of domain scores, which showed that most participants were classified in low or moderate categories across domains, particularly for situational use and social acceptability. This suggests that while vaping is present and increasing, it may not yet be fully normalized across all behavioral contexts within this population. However, the relatively high proportion of participants in moderate categories especially in media influence and perceived utility indicates a transitional phase in which attitudes and behaviors may be shifting. This pattern is consistent with diffusion of innovation theory, where adoption of new behaviors progresses through stages of awareness, interest, and eventual normalization [32].

From a public health perspective, these findings have important implications. The consistent pattern of higher domain scores among males suggests that interventions targeting vaping behavior may need to be sex-specific, with greater emphasis on addressing perceived benefits and marketing influence among men. At the same time, the presence of moderate levels of influence among females indicates a potential window for preventive interventions before further normalization occurs. Educational campaigns that address misperceptions regarding harm and challenge pro-vaping narratives may be particularly effective in this context.

Several limitations should be considered when interpreting these findings. First, the use of a non-probability snowball sampling approach limits the generalizability of the results, as the sample may not be representative of all young adults in Jeddah or Saudi Arabia. Second, the cross-sectional design precludes causal inference, and the observed associations cannot establish temporal relationships between exposure and outcome. Third, data were self-reported and may be subject to recall bias or social desirability bias, particularly given the sensitive nature of tobacco use among females in some cultural contexts. Finally, the reliance on complete-case analysis may introduce selection bias if excluded respondents differed systematically from those included in the analysis. Despite these limitations, this study provides novel, multidimensional evidence on sex differences in vaping-related perceptions and behaviors within the Saudi context. By integrating behavioral, perceptual, and social domains, the findings offer a more comprehensive understanding of vaping beyond simple prevalence measures. Future research should build on these findings using longitudinal designs and representative sampling approaches to better understand temporal trends and causal pathways.

## 5. Conclusions

In this cross-sectional study of young adults in Jeddah, Saudi Arabia, clear sex-based differences were observed across multiple vaping-related domains. Males demonstrated higher engagement and more favorable perceptions of e-cigarettes, whereas females consistently reported lower endorsement across behavioral, perceptual, and social domains. These findings suggest that vaping behaviors are shaped by underlying differences in perception and social influence rather than prevalence alone. Targeted, sex-specific public health interventions addressing perceived benefits and marketing exposure are warranted, particularly in the context of evolving social norms.

## Figures and Tables

**Table 1:** Univariable analysis of participant characteristics and domain scores by sex (N = 388). All proportions expressed as % of total cohort with 95% Wilson confidence intervals. Effect size: Cramér’s V. Reference: Male.

Variable/Domain	Category	Male n (%)	Male 95% Wilson CI	Female n (%)	Female 95% Wilson CI	Total n (%)	*p* Value	V
**Total participants**	N	228 (58.8%)	54.2–63.3%	160 (41.2%)	36.7–45.8%	388 (100%)	—	—
**Situational Use & Behavioral Context**								
	Low	124 (32.0%)	27.5–36.8%	109 (28.1%)	23.9–32.8%	233 (60.1%)	**0.016**	0.146
	Medium	43 (11.1%)	8.3–14.6%	17 (4.4%)	2.8–6.9%	60 (15.5%)		
	High	61 (15.7%)	12.4–19.7%	34 (8.8%)	6.3–12.0%	95 (24.5%)		
**Perceived Utility & Functional Benefits**								
	Low	86 (22.2%)	18.3–26.6%	92 (23.7%)	19.7–28.2%	178 (45.9%)	**<0.001**	0.195
	Medium	103 (26.5%)	22.4–31.2%	49 (12.6%)	9.7–16.3%	152 (39.2%)		
	High	39 (10.1%)	7.4–13.4%	19 (4.9%)	3.2–7.5%	58 (14.9%)		
**Risk Perception & Sensory Evaluation**								
	Low	74 (19.1%)	15.5–23.3%	91 (23.5%)	19.5–27.9%	165 (42.5%)	**<0.001**	0.254
	Medium	80 (20.6%)	16.9–24.9%	28 (7.2%)	5.0–10.2%	108 (27.8%)		
	High	74 (19.1%)	15.5–23.3%	41 (10.6%)	7.9–14.0%	115 (29.6%)		
**Social Acceptability & Norms**								
	Low	137 (35.3%)	30.7–40.2%	117 (30.2%)	25.8–34.9%	254 (65.5%)	**0.022**	0.140
	Medium	71 (18.3%)	14.8–22.5%	36 (9.3%)	6.8–12.6%	107 (27.6%)		
	High	20 (5.2%)	3.4–7.8%	7 (1.8%)	0.9–3.7%	27 (7.0%)		
**Media & Marketing Influence**								
	Low	73 (18.8%)	15.2–23.0%	94 (24.2%)	20.2–28.7%	167 (43.0%)	**<0.001**	0.260
	Medium	155 (39.9%)	35.2–44.9%	66 (17.0%)	13.6–21.1%	221 (57.0%)		
**Sociodemographic characteristics**								
**Monthly income**	Under 2000 SAR	137 (35.3%)	30.7–40.2%	105 (27.1%)	22.9–31.7%	242 (62.4%)	0.304	0.078
	Above 2000 SAR	60 (15.5%)	12.2–19.4%	41 (10.6%)	7.9–14.0%	101 (26.0%)		
	No income	31 (8.0%)	5.7–11.1%	14 (3.6%)	2.2–6.0%	45 (11.6%)		
**Marital status**	Single	223 (57.5%)	52.5–62.3%	148 (38.1%)	33.4–43.1%	371 (95.6%)	**0.024**	0.115
	Married/Other	5 (1.3%)	0.6–3.0%	12 (3.1%)	1.8–5.3%	17 (4.4%)		

N = 388 total participants (Male n = 228, 58.8%; Female n = 160, 41.2%). Values are n (%) with 95% Wilson confidence intervals. Wilson CIs outperform Wald CIs for proportions near 0 or 1. All percentages are of the total cohort (N = 388) unless stated. Cramér’s V quantifies effect size for chi-square tests (V ≥ 0.10 small, ≥0.30 moderate, ≥0.50 large). *p* values from Pearson chi-square test comparing sex distributions across all categories per domain. Domain classification: Low = below threshold, Medium = intermediate, High = above threshold response. Bold *p* values denote statistical significance (*p* < 0.05). Media & Marketing Influence: High category not reported (data not available in source table). Marital status: Married/Other category includes all non-single participants (n = 17, 4.4%). SAR = Saudi Arabian Riyal; CI = confidence interval; V = Cramér’s V effect size.

**Table 2:** Ordinal logistic regression of domain classification by sex, income, and marital status (N = 388). Reference groups: Male sex|Monthly income > 2000 SAR|Married · Outcome ordered: Low → Medium → High. aOR < 1.0 = lower odds of higher classification · aOR > 1.0 = higher odds of higher classification.

Outcome Domain	Predictor (Reference Group)	aOR	95% CI Lower	95% CI Upper	*p* Value
**Situational Use & Behavioral Context**					
**Reference groups: Male sex|Income > 2000 SAR|Married · Outcome ordered: Low → Medium → High**					
	Female vs. Male (ref)	0.62	0.45	0.86	**0.004**
	<2000 SAR vs. >2000 SAR (ref)	0.71	0.52	0.97	**0.032**
	No income vs. >2000 SAR (ref)	0.88	0.55	1.41	0.601
	Single vs. Married (ref)	1.29	0.64	2.61	0.480
**Perceived Utility & Functional Benefits**					
**Reference groups: Male sex|Income >2000 SAR|Married · Outcome ordered: Low → Medium → High**					
	Female vs. Male (ref)	0.58	0.41	0.81	**0.002**
	<2000 SAR vs. >2000 SAR (ref)	0.66	0.48	0.91	**0.011**
	No income vs. >2000 SAR (ref)	0.93	0.58	1.49	0.760
	Single vs. Married (ref)	1.18	0.59	2.36	0.640
**Risk Perception & Sensory Evaluation**					
**Reference groups: Male sex|Income > 2000 SAR|Married · Outcome ordered: Low → Medium → High**					
	Female vs. Male (ref)	0.54	0.39	0.75	**<0.001**
	<2000 SAR vs. >2000 SAR (ref)	0.61	0.45	0.83	**0.002**
	No income vs. >2000 SAR (ref)	0.79	0.50	1.25	0.310
	Single vs. Married (ref)	1.22	0.61	2.45	0.570
**Social Acceptability & Norms**					
***Reference groups: Male sex|Income > 2000 SAR|Married · Outcome ordered: Low*** ***→*** ***Medium*** ***→*** ***High***					
	Female vs. Male (ref)	0.63	0.45	0.88	**0.007**
	<2000 SAR vs. >2000 SAR (ref)	0.74	0.54	1.01	0.058
	No income vs. >2000 SAR (ref)	1.05	0.66	1.68	0.830
	Single vs. Married (ref)	1.35	0.67	2.72	0.400
**Media & Marketing Influence**					
**Reference groups: Male sex|Income > 2000 SAR|Married · Outcome ordered: Low → Medium → High**					
	Female vs. Male (ref)	0.49	0.35	0.69	**<0.001**
	<2000 SAR vs. >2000 SAR (ref)	0.59	0.43	0.81	**0.001**
	No income vs. >2000 SAR (ref)	0.91	0.57	1.44	0.680
	Single vs. Married (ref)	1.48	0.74	2.98	0.260
**Total Score (Categorized—composite outcome)**					
**Reference groups: Male sex|Income > 2000 SAR|Married · Outcome ordered: Low → Medium → High**					
	Female vs. Male (ref)	0.56	0.40	0.79	**0.001**
	<2000 SAR vs. >2000 SAR (ref)	0.63	0.46	0.86	**0.004**
	No income vs. >2000 SAR (ref)	0.97	0.61	1.55	0.900
	Single vs. Married (ref)	1.41	0.70	2.83	0.330

aOR = adjusted odds ratio; CI = 95% confidence interval. Models fitted using ordinal logistic regression (proportional odds). Each model includes all four predictors simultaneously (sex, income, marital status). aOR < 1.0 = lower odds of higher classification category; aOR > 1.0 = higher odds. N = 388. SAR = Saudi Arabian Riyal.

## Data Availability

The original contributions presented in this study are included in the article. Further inquiries can be directed to the corresponding authors.

## Abbreviations

PATH	Population Assessment of Tobacco and Health.
SAR	Saudi Arabian Riyal.
aOR	Adjusted Odds Ratio.
CI	Confidence Interval.
V	Cramér’s V Effect Size.

## References

[1] Pan L., Morton J., Mbulo L., Dean A., Ahluwalia I.B. (2022). Electronic cigarette use among adults in 14 countries: A cross-sectional study. eClinicalMedicine.

[2] Gebeyehu N.A., Gelaw K.A., Atalay Y.A., Walle B.G., Gesese M.M., Admass B.A., Birhan B., Geberemariam A.E., Alemu B.W., Shewangashaw N.E. (2025). Global prevalence of E-cigarette use among students: Systematic review and meta-analysis. PLoS ONE.

[3] Alhomoud F.K., Almuhayshi A., Altarouti R., Abushaheen T., Alhomoud F., Alotaibi N., Alsugeir D., Alamer K.A., Alqarni Y., Alfageh B. (2025). Electronic cigarette use in Saudi Arabia: A cross-sectional study on emerging trends and public health concerns. Front. Public Health.

[4] Alzahrani B.M. (2025). Social Determinants of Health and Use of E-Cigarettes Among Saudi Adult Users in Riyadh City, Saudi Arabia: A Cross-Sectional Study. Master’s Thesis.

[5] Alahmadi S.M., Al-Zalabani A.H. (2025). Patterns and factors associated with e-cigarette initiation and transition among university students in Al-Madinah City, Saudi Arabia: A cross-sectional study. Healthcare.

[6] Aloufi N., Alhamawi R.M., Alalwani S.N., Alrefaei W.K., Aljohani H.A., Ali M.M., Alahmadi F.H. (2025). Prevalence of e-cigarette users in the Medina region of Saudi Arabia. Tob. Induc. Dis..

[7] Alhajj M.N., Al-Maweri S.A., Folayan M.O., Halboub E., Khader Y., Omar R., Amran A.G., Al-Batayneh O.B., Celebić A., Persic S. (2022). Knowledge, beliefs, attitude, and practices of E-cigarette use among dental students: A multinational survey. PLoS ONE.

[8] Alluhaidan R., Babutain A., Alharbi M., Fiala L. (2024). Knowledge, perception, and use of vape among the Saudi population in Riyadh, Saudi Arabia. Eur. J. Med. Health Res..

[9] Al Chami R., Youssef M.H. (2025). Challenging patriarchy: The transformation of women’s roles under Saudi Arabia’s Vision 2030. Impact of Patriarchy and Gender Stereotypes on Working Women: Exploring Its Past, Present and Future.

[10] Macias-Alonso I., Kim H., González A.L. (2023). Self-driven women: Gendered mobility, employment, and the lift of the driving ban in Saudi Arabia. Gend. Place Cult..

[11] Alalweet R.M.U. (2025). Relationship of Media and Entertainment Exposure to Tobacco Use Among Teenagers in Saudi Arabia: A National Study. Master’s Thesis.

[12] Ramadan M., Alsiary R.A., Aboalola D.A., Aouabdi S. (2025). Cross-country comparison of bans on internet tobacco advertising, and search interest in vaping products. Tob. Use Insights.

[13] Murray J.M., Sánchez-Franco S.C., Sarmiento O.L., Kimbrough E.O., Tate C., Montgomery S.C., Kumar R., Dunne L., Ramalingam A., Krupka E.L. (2025). Moderators of peer influence effects for adolescents’ smoking and vaping norms and outcomes in high and middle-income settings. Front. Psychol..

[14] Alshahrani N.Z., Alarifi A.M., Qarah M., Almalki S., Alshammari W.H., Alnahdi R.S., Shukri A.K., Alshahrani B.A., Alamri S.A.M., Ghazwani S.M. (2026). Prevalence and correlates of ever and current dual use of cigarettes and e-cigarettes among adolescents in Saudi Arabia. Intern. Emerg. Med..

[15] Ashour A.M. (2023). Use of vaping as a smoking cessation aid: A review of clinical trials. J. Multidiscip. Healthc..

[16] Sargent J.D., Halenar M.J., Edwards K.C., Woloshin S., Schwartz L., Emond J., Tanski S., Taylor K.A., Pierce J.P., Liu J. (2022). Tobacco use and respiratory symptoms among adults: Findings from the longitudinal Population Assessment of Tobacco and Health (PATH) study 2014–2016. Nicotine Tob. Res..

[17] Rozenkowska K. (2023). Theory of planned behavior in consumer behavior research: A systematic literature review. Int. J. Consum. Stud..

[18] Ghaferi A.A., Schwartz T.A., Pawlik T.M. (2021). STROBE reporting guidelines for observational studies. JAMA Surg..

[19] Hyland A., Ambrose B.K., Conway K.P., Borek N., Lambert E., Carusi C., Taylor K., Crosse S., Fong G.T., Cummings K.M. (2017). Design and methods of the Population Assessment of Tobacco and Health (PATH) Study. Tob. Control.

[20] East K., Hitchman S.C., Bakolis I., Williams S., Cheeseman H., Arnott D., McNeill A. (2018). The association between smoking and electronic cigarette use in a cohort of young people. J. Adolesc. Health.

[21] Leventhal A.M., Strong D.R., Kirkpatrick M.G., Unger J.B., Sussman S., Riggs N.R., Stone M.D., Khoddam R., Samet J.M., Audrain-McGovern J. (2015). Association of electronic cigarette use with initiation of combustible tobacco product smoking in early adolescence. JAMA.

[22] Bold K.W., Kong G., Cavallo D.A., Camenga D.R., Krishnan-Sarin S. (2016). Reasons for trying e-cigarettes and risk of continued use. Pediatrics.

[23] Altman D.G., Royston P. (2006). The cost of dichotomising continuous variables. BMJ.

[24] De Vito R., Stephenson B., Sotres-Alvarez D., Siega-Riz A.-M., Mattei J., Parpinel M., Peters B.A., Bainter S.A., Daviglus M.L., Van Horn L. (2025). Identifying and characterizing shared and ethnic background site-specific dietary patterns in the Hispanic Community Health Study/Study of Latinos (HCHS/SOL). Nutr. J..

[25] Rayes B.T., Alalwan A., AbuDujain N.M., Darraj A., Alammar M.A., Jradi H. (2023). Prevalence, trends, and harm perception associated with e-cigarettes and vaping among adolescents in Saudi Arabia. Arch. Clin. Biomed. Res..

[26] Jackson S.E., Tattan-Birch H., Shahab L., Brown J. (2024). Trends in long term vaping among adults in England 2013-23: Population-based study. BMJ.

[27] Vahratian A., Briones E.M., Jamal A., Marynak K.L. (2025). Electronic cigarette use among adults in the United States 2019–2023. NCHS Data Brief.

[28] Lucherini M., Hill S., Smith K. (2020). Inequalities, harm reduction and non-combustible nicotine products: A meta-ethnography of qualitative evidence. BMC Public Health.

[29] Malt L., Verron T., Cahours X., Guo M., Weaver S., Walele T., O’Connell G. (2020). Perception of the relative harm of electronic cigarettes compared to cigarettes amongst US adults from 2013 to 2016: Analysis of the Population Assessment of Tobacco and Health (PATH) study data. Harm Reduct. J..

[30] Weber A.M., Cislaghi B., Meausoone V., Abdalla S., Mejía-Guevara I., Loftus P., Hallgren E., Seff I., Stark L., Victora C.G. (2019). Gender norms and health: Insights from global survey data. Lancet.

[31] Mahlakaarto E.K., Suanse Y. (2024). How Women’s Consumer Identity Is Shaped by Social Media and Influencers in the Beauty and Lifestyle Industry. Master’s Thesis.

[32] Zou W., Wang X., Yang N., Ni X., Zhao Z., Meng R., Ma H. (2024). The intention of college students to use electronic cigarettes: A study based on the theory of innovation diffusion. Tob. Induc. Dis..

